# Character gated recurrent neural networks for Arabic sentiment analysis

**DOI:** 10.1038/s41598-022-13153-w

**Published:** 2022-06-13

**Authors:** Eslam Omara, Mervat Mousa, Nabil Ismail

**Affiliations:** 1 Agriculture Directorate, Cairo Governorate, Cairo, Egypt; 2 Faculty of Electronic Engineering, Menofia University, Menouf, Egypt

**Keywords:** Electrical and electronic engineering, Learning algorithms, Neural encoding

## Abstract

Sentiment analysis is a Natural Language Processing (NLP) task concerned with opinions, attitudes, emotions, and feelings. It applies NLP techniques for identifying and detecting personal information from opinionated text. Sentiment analysis deduces the author's perspective regarding a topic and classifies the attitude polarity as positive, negative, or neutral. In the meantime, deep architectures applied to NLP reported a noticeable breakthrough in performance compared to traditional approaches. The outstanding performance of deep architectures is related to their capability to disclose, differentiate and discriminate features captured from large datasets. Recurrent neural networks (RNNs) and their variants Long-Short Term Memory (LSTM), Gated Recurrent Unit (GRU), Bi-directional Long-Short Term Memory (Bi-LSTM), and Bi-directional Gated Recurrent Unit (Bi-GRU) architectures are robust at processing sequential data. They are commonly used for NLP applications as they—unlike RNNs—can combat vanishing and exploding gradients. Also, Convolution Neural Networks (CNNs) were efficiently applied for implicitly detecting features in NLP tasks. In the proposed work, different deep learning architectures composed of LSTM, GRU, Bi-LSTM, and Bi-GRU are used and compared for Arabic sentiment analysis performance improvement. The models are implemented and tested based on the character representation of opinion entries. Moreover, deep hybrid models that combine multiple layers of CNN with LSTM, GRU, Bi-LSTM, and Bi-GRU are also tested. Two datasets are used for the models implementation; the first is a hybrid combined dataset, and the second is the Book Review Arabic Dataset (BRAD). The proposed application proves that character representation can capture morphological and semantic features, and hence it can be employed for text representation in different Arabic language understanding and processing tasks.

## Introduction

Natural language processing considers many tasks to analyze the text structure and understand its semantics. The extracted syntactic and semantic information is then exploited for a higher-level task. Examples of NLP tasks are Part-of-speech Tagging (POS)^1^, Chunking or shallow parsing^2^, Parsing^3^, Semantic role labeling (SRL)^1^, Named entity recognition (NER)^1^, Word-sense disambiguation^4^, Anaphora resolution (pronoun resolution)^5^, Sentence classification^6^, Sentiment analysis^7^, Emotion detection (ED)^8,9^, Document classification^10^, Text summarization^11^, Machine translation^3^, and Question answering (QA)^2^. Natural language processing tasks can be categorized according to the revealed information as^1,2^:

- Syntactic tasks as part-of-speech tagging, chunking, and parsing.
- Semantic tasks include sentiment analysis, emotion detection, document classification, text summarization, machine translation, question answering, sentence classification, word-sense disambiguation, semantic role labelling, named entity recognition, and anaphora resolution.

NLP tasks were investigated by applying statistical and machine learning techniques. Recently, deep learning (DL) structures are extensively used in NLP. Deep learning models can identify and learn features from raw data, and they registered superior performance in various fields^12^. In addition to natural language processing, DL were employed in computer vision, handwriting recognition, speech recognition, object detection, cancer detection, biological image classification, face recognition, stock market analysis, and many others^13^.

Deep learning applies a variety of architectures capable of learning features that are internally detected during the training process. RNNs are deep learning architectures commonly used for sequence modelling. The recurrence connection in RNNs supports the model to memorize dependency information included in the sequence as context information in natural language tasks^14^. And hence, RNNs can account for words order within the sentence enabling preserving the context^15^. Unlike feedforward neural networks that employ the learned weights for output prediction, RNN uses the learned weights and a state vector for output generation^16^. Long-Short Term Memory (LSTM), Gated Recurrent Unit (GRU), Bi-directional Long-Short Term Memory (Bi-LSTM), and Bi-directional Gated Recurrent Unit (Bi-GRU) are variants of the simple RNN. The variants are based on the notion of gates^16,17^.

Contrary to RNN, gated variants are capable of handling long term dependencies. Also, they can combat vanishing and exploding gradients by the gating technique^14^. Bi-directional recurrent networks can handle the case when the output is predicted based on the input sequence's surrounding components^18^. LSTM is the most widespread DL architecture applied to NLP as it can capture far distance dependency of terms^15^. GRUs implemented in NLP tasks are more appropriate for small datasets and can train faster than LSTM^17^.

In addition to gated RNNs, Convolutional Neural Network (CNN) is another common DL architecture used for feature detection in different NLP tasks. For example, CNNs were applied for SA in deep and shallow models based on word and character features^19^. Moreover, hybrid architectures—that combine RNNs and CNNs—demonstrated the ability to consider the sequence components order and find out the context features in sentiment analysis^20^. These architectures stack layers of CNNs and gated RNNs in various arrangements such as CNN-LSTM, CNN-GRU, LSTM-CNN, GRU-CNN, CNN-Bi-LSTM, CNN-Bi-GRU, Bi-LSTM-CNN, and Bi-GRU-CNN. Convolutional layers help capture more abstracted semantic features from the input text and reduce dimensionality. RNN layers capture the gesture of the sentence from the dependency and order of words.

Meanwhile, many customers create and share content about their experience on review sites, social channels, blogs etc. The valuable information in the authors tweets, reviews, comments, posts, and form submissions stimulated the necessity of manipulating this massive data. The revealed information is an essential requirement to make informed business decisions. Sentiment analysis is a crucial NLP task that aims at studying and understanding personal emotions, behaviours, opinions, feelings, and assessments of various targets such as services, facilities, products, problems, items, firms, occasions, topics, and public figures^8,19^. Understanding individuals sentiment is the basis of understanding, predicting, and directing their behaviours. By applying NLP techniques, SA detects the polarity of the opinioned text and classifies it according to a set of predefined classes. Statistical, machine learning and deep learning methodologies applied for SA performance improvement tackled problems such as capturing context information, considering dialectical language, handling social media text's unique nature and identifying the sentiment holder.

In the Arabic language, the character form changes according to its location in the word. It can be written connected or disconnected at the end, placed within the word, or found at the beginning. Besides, diacritics or short vowels control the word phonology and alter its meaning. These characteristics propose challenges to word embedding and representation^21^. Further challenges for Arabic language processing are dialects, morphology, orthography, phonology, and stemming^21^. In addition to the Arabic nature related challenges, the efficiency of word embedding is task-related and can be affected by the abundance of task-related words^22^. Therefore, a convenient Arabic text representation is required to manipulate these exceptional characteristics. Most implementations of LSTMs and GRUs for Arabic SA employed word embedding to encode words by real value vectors. Besides, the common CNN-LSTM combination applied for Arabic SA used only one convolutional layer and one LSTM layer.

Up to the available knowledge, the performance of deep LSTM, GRU, Bi-LSTM, and Bi-GRU has not been investigated in Arabic language SA using character representation. Furthermore, deep hybrid combinations such as CNN-LSTM, CNN-GRU, LSTM-CNN, GRU-CNN, CNN-Bi-LSTM, CNN-Bi-GRU, Bi-LSTM-CNN, and Bi-GRU-CNN have not been studied or compared. Therefore, the contributions of the proposed work are:

- Four architectures of deep LSTM, GRU, Bi-LSTM and Bi-GRU are investigated based on character features for Arabic sentiment analysis.
- Eight deep hybrid CNN-LSTM, CNN-GRU, LSTM-CNN, GRU-CNN, CNN-Bi-LSTM, CNN-Bi-GRU, Bi-LSTM-CNN, and Bi-GRU-CNN structures that merge layers of different architectures are also implemented and validated.
- The presented deep networks are tested on two datasets; the first is a hybrid dataset that was built from multiple available datasets dedicated to Arabic SA. The second and the benchmarking dataset is the Arabic book reviews dataset (BRAD).
- The proposed application examines the ability of deep networks to detect discriminating features from data represented at the character level.
- Extensive empirical analysis of the predictive performance of the twelve networks using the two datasets is conducted to find out the architectures that best fit the low-level representation.

The remainder of the paper is organized as follows: the “Sentiment analysis” section explains notions, concepts, and definitions related to sentiment analysis and the “Feature representation” section discusses the approaches commonly used to represent features for NLP tasks. The literature review is introduced in the “Related work” section. The “Applied models” section clarifies in details the structure and settings of the implemented networks. Results invistigation and empirical analysis are proposed in the “Experiments and results” section. Finally, the concluded results and further future work are declared in the “Conclusion” section.

## Sentiment analysis

SA research depends on data originating from social media, such as tweets, reviews, and comments. Lately, medical services, stock market, and human emotions were discussed while early topics included reviews, product features, and elections^23^. Sentiment analysis has been studied at multiple granularity levels: document, sentence, and aspect. Each opinionated text is considered one unit and assigned a positive, negative, or neutral polarity at the document level. The document holds an opinion regarding a single entity and has one opinion holder. Opinions that maintain multiple entities assessment cannot be analyzed using this level^6,24^. Sentence level SA begins with determining if the sentence expresses an opinion or not (subjective or objective). This step is known as subjectivity classification. Next, the sentiment orientation of emotional sentences is identified by multi-class or binary classification. The multi-class classification assigns a positive, negative, or neutral category to subjective sentences, whereas the binary type considers only positive and negative classes^6,25^. A more fine-grained SA is the aspect level or phrase level that defines the quintuple (Object, Aspect, Sentiment Orientation, Opinion Holder, Time) components of an opinion concerning an entity or an entity feature. It is also called feature-based sentiment analysis. An argument about an object may hold a positive orientation regarding a characteristic and a negative orientation regarding another characteristic, so it is not positive or negative for the whole entity^24,25^.

Sentiment analysis is generally applied using three approaches. Most machine learning algorithms applied for SA are mainly supervised approaches such as Support Vector Machine (SVM), Naïve Bayes (NB), Artificial Neural Networks (ANN), and K-Nearest Neighbor (KNN)^26^. A large labelled dataset is required to train a robust classifier. But, large pre-annotated datasets are usually unavailable and extensive work, cost, and time are consumed to annotate the collected data. Lexicon based approaches use sentiment lexicons that contain words and their corresponding sentiment scores. The corresponding value identifies the word polarity (positive, negative, or neutral). These approaches do not use labelled datasets but require wide-coverage lexicons that include many sentiment holding words. Dictionaries are built by applying corpus-based or dictionary-based approaches^6,26^. The lexicon approaches are popularly used for Modern Standard Arabic (MSA) due to the lack of vernacular Arabic dictionaries^6^. Sentiment polarities of sentences and documents are calculated from the sentiment score of the constituent words/phrases. Most techniques use the sum of the polarities of words and/or phrases to estimate the polarity of a document or sentence^24^. The lexicon approach is named in the literature as an unsupervised approach because it does not require a pre-annotated dataset. It depends mainly on the mathematical manipulation of the polarity scores, which differs from the unsupervised machine learning methodology. The hybrid approaches (Semi-supervised or weakly supervised) combine both lexicon and machine learning approaches. It manipulates the problem of labelled data scarcity by using lexicons to evaluate and annotate the training set at the document or sentence level. Un-labelled data are then classified using a classifier trained with the lexicon-based annotated data^6,26^.

## Feature representation

Processing unstructured data such as text, images, sound records, and videos are more complicated than processing structured data. The difficulty of capturing semantics and concepts of the language from words proposes challenges to the text processing tasks. A document can not be processed in its raw format, and hence it has to be transformed into a machine-understandable representation^27^. Selecting the convenient representation scheme suits the application is a substantial step^28^. The fundamental methodologies used to represent text data as vectors are Vector Space Model (VSM) and neural network-based representation. Text components are represented by numerical vectors which may represent a character, word, paragraph, or the whole document. VSM can be formulated by many approaches^28,29^.

Binary representation is an approach used to represent text documents by vectors of a length equal to the vocabulary size. Documents are quantized by One-hot encoding to generate the encoding vectors^30^. The representation does not preserve word meaning or order, so similar words cannot be distinguished from entirely different worlds. One-hot encoding of a document corpus is a vast sparse matrix resulting in a high dimensionality problem^28^. This representation is referred to as discrete or local representation^29^.

The bag of Word (BOW) approach constructs a vector representation of a document based on the term frequency. BOW is widely speared for text classification applications^27^. However, a drawback of BOW representation is that word order is not preserved, resulting in losing the semantic associations between words. Another limitation is that each word is represented as a distinct dimension. The representation vectors are sparse, with too many dimensions equal to the corpus vocabulary size^31^. Also, there exist many cases of polysemous and homonymous. Polysemy refers to the presence of many possible meanings for a word. Homonymy means the existence of two or more words with the same spelling or pronunciation but different meanings and origins. Words with different semantics and the same spelling have the same representation. And synonym words with different spelling have completely different representations^28,29^. Representing documents based on the term frequency does not consider that common words have higher occurrence than other words, and so the corresponding dimensions are defined by much higher values than rare but discriminating words. Term weighting techniques are applied to assign appropriate weights to the relevant terms to handle such problems. Term Frequency-Inverse Document Frequency (TF-IDF) is a weighting schema that uses term frequency and inverse document frequency to discriminate items^29^.

Bag-Of-N-Grams (BONG) is a variant of BOW where the vocabulary is extended by appending a set of N consecutive words to the word set. The N-words sequences extracted from the corpus are employed as enriching features. But, the number of words selected for effectively representing a document is difficult to determine^27^. The main drawback of BONG is more sparsity and higher dimensionality compared to BOW^29^. Bag-Of-Concepts is another document representation approach where every dimension is related to a general concept described by one or multiple words^29^.

Alternatively, words can be quantized by a distributed representation. Each word is assigned a continuous vector that belongs to a low-dimensional vector space. Neural networks are commonly used for learning distributed representation of text, known as word embedding^27,29^. Popular neural models used for learning word embedding are Continuous Bag-Of-Words (CBOW)^32^, Skip-Gram^32^, and GloVe^33^ embedding. In CBOW, word vectors are learned by predicting a word based on its context. A context is a predefined number of words around the expected word. Skip-Gram follows a reversed strategy as it predicts the context words based on the centre word. GloVe uses the vocabulary words co-occurrence matrix as input to the learning algorithm where each matrix cell holds the number of times by which two words occur in the same context. A discriminant feature of word embedding is that they capture semantic and syntactic connections among words. Embedding vectors of semantically similar or syntactically similar words are close vectors with high similarity^29^.

Learning word embedding depends on a distributional assumption which supposes that words with similar meanings occur in similar contexts and hence they have comparable distributions^27^. Relying on word co-occurrence may place antonymous words near each other in the vector space, which can be a drawback of word embedding. For example, “good and bad” may be assigned close vectors because they often appear in similar contexts. The efficiency of word embedding may be affected by such cases, especially in tasks like SA^29^.

In the proposed investigation, the SA task is inspected based on character representation, which reduces the vocabulary set size compared to the word vocabulary. Besides, the learning capability of deep architectures is exploited to capture context features from character encoded text.

## Related work

Recurrent neural networks (RNNs) and their gated variants, Long-Short Term Memory (LSTM) and Gated Recurrent Unit (GRU), have been applied in different NLP tasks such as text generation, sentiment analysis, machine translation, question answering, and summarization. The applications exploit the capability of RNNs and gated RNNs to manipulate inputs composed of sequences of words or characters^17,34^. RNNs process chronological sequence in both input and output, or only one of them. According to the investigated problem, RNNs can be arranged in different topologies^16^. In addition to the homogenous arrangements composed of one type of deep learning networks, there are hybrid architectures combine different deep learning networks. The hybrid architectures avail from the outstanding characteristic of each network type to empower the model.

CNN, LSTM, Bi-LSTM, and GRU were implemented using word and character embedding for sentiment categorization^34^. Bi-LSTM showed the best performance using the word embedding, whereas CNN reported the best performance using the character embedding. The results were further enhanced by combining the features disclosed from character CNN and word Bi-LSTM in a hybrid model. The integrated features were fed to the classification layer for polarity identification, and the model showed more boosted performance. Also, CNN, RNN, LSTM, GRU, and CNN-LSTM were tested for sentiment analysis of product reviews and based on word embedding, the CNN-LSTM architecture registered the highest performance^35^. LSTM reported the second-highest performance. It was highlighted that LSTM is efficient at NLP tasks. Shallow LSTM, GRU, Bi-LSTM, and Bi-GRU were trained and compared using the Amazon review corpus^36^. Results reported that bi-directional structures reached higher performance compared to unidirectional versions. Additionally, GRU trained faster and outperformed LSTM.

A comparative study was conducted applying multiple deep learning models based on word and character features^37^. Three CNN and five RNN networks were implemented and compared on thirteen reviews datasets. One, nine, and twenty-nine layers CNN models were implemented. Also, RNN, LSTM, GRU, Bi-LSTM, and Bi-GRU architectures were tested. Although the thirteen datasets included reviews, the deep models performance varied according to the domain and the characteristics of the dataset. Based on word-level features Bi-LSTM, GRU, Bi-GRU, and the one layer CNN reached the highest performance on numerous review sets, respectively. Based on character level features, the one layer CNN, Bi-LSTM, twenty-nine layers CNN, GRU, and Bi-GRU achieved the best measures consecutively. A sentiment categorization model that employed a sentiment lexicon, CNN, and Bi-GRU was proposed in^38^. Sentiment weights calculated from the sentiment lexicon were used to weigh the input embedding vectors. The CNN-Bi-GRU network detected both sentiment and context features from product reviews better than the networks that applied only CNN or Bi-GRU.

For Arabic SA, a lexicon was combined with RNN to classify sentiment in tweets^39^. An RNN network was trained using feature vectors computed using word weights and other features as percentage of positive, negative and neutral words. RNN, SVM, and L2 Logistic Regression classifiers were tested and compared using six datasets. In addition, LSTM models were widely applied for Arabic SA using word features and applying shallow structures composed of one or two layers^15,40–42^, as shown in Table 1.

**Table 1:** Arabic sentiment analysis using RNNs and gated RNN.

	^39^	^15^	^40^	^41^	^42^	^47^	^48^	^49^	^43^	^44^	^45^	^46^	^14^	^50^
Model	RNN	LSTM DNN	LSTM CNN RCNN	LSTM CNN	LSTM	LSTM Bi-LSTM	CNN Bi-LSTM	Bi-LSTM LSTM CNN	CNN LSTM CNN-LSTM	CNN-LSTMs	CNN-LSTM	CNN-LSTM	LSTM GRU Bi-LSTM Bi-GRU	GRU
Layers		Two layers	One layer	One layer	One layer	One layer Two layers	One layer	One layer	One layer Three layers	Conv. layer LSTM layer	Conv. layer Two LSTM layers	Conv. layer LSTM layer	One layer	One layer
Features	Word weight	Binary vectors	Word embedding	Word embedding	Word embedding	Word embedding	Word embedding	Word embedding	Word embedding	Character Character N-gram Word	Word embedding	Word embedding	Emojis	Word embedding
Dataset	ASTD 9174 tweets MASTD 1850 tweets ArSAS 19,762 tweets GS 4191 tweets Syrian 2000 tweets ArTwitter 2000 tweets	LABR 63,000 reviews	40,000 tweets	MSAC 2000 reviews SemEval-2017 task 4 2000 tweets and comments	LABR 16,448 reviews	TSAC 17,069 comments	ASTD 10,000 tweets	ASTD 1589 tweets ArTwitter 1951tweets LABR 16,448 reviews MPQA 9996 news articles Multi-domain resource 31,598 reviews Main-AHS 2026 tweets	ASTD 10,000 tweets ArTwitter 2000 tweets	Main-AHS 2026 tweets Sub-AHS 1732 tweets Ar-Twitter 2000 tweets ASTD 2479 tweets	Ar-Twitter 2000 tweets ASTD 10,000 tweets LABR 63,000 reviews	SemEval 2017 task 4-A 9655 tweets ASTD 10,000 tweets ArSAS 17,784 tweets	Tweets YouTube comments 2091	Religious hate 6600 tweets
Preprocessing	Tokenization Normalization Stemming Deleting non-Arabic words, numbers, URLs, users mentions, hashtags, stop words, names Detecting intensification, emoji, idioms, negation	Tokenization Stemming Removing punctuation marks, stop-words, tab space, blank space	Removing special characters, none Arabic letters, diacritics, elongation Normalization Manual correction	Tokenization Normalization Stemming Deleting stop words	Normalization Stemming Removing diacritics, repetition, punctuations, stop words, non-Arabic words		Normalization Removing numbers, punctuation symbols, elongation, diacritics Replace emoticons with emoji Tokenize emoji	Tokenization Normalization Stemming Removing stop words, punctuations, Latin characters, digits	Deleting non-Arabic symbols, dialectical marks, punctuation marks, tatweel, duplicate character	Tokenization (character, character 5-g, word)	Normalization Stemming Lemmatization Removing stop words, duplicated letters Filtering non-Arabic words Spelling correction	Normalization Removing elongation, unknown characters, diacritics, punctuation	Normalization Removing tweets with no Emojis	Normalization Handling elongation Deleting stop words, diacritics, punctuations, emojis, tatweel, one-letter words, non-Arabic characters

LSTMs were used for classifying short tweets and lengthy reviews. It was noted that LSTM outperformed CNN in SA when used in a shallow structure based on word features. Applying the data shuffling augmentation technique enhanced the LSTM model performance^40^. In another context, the impact of morphological features on LSTM and CNN performance was tested by applying different preprocessing steps steps such as stop words removal, normalization, light stemming and root stemming^41^. It was reported that preprocessing steps that eliminate text noise and reduce distortions in the feature space affect the classification performance positively. Whilst, preprocessing actions that cause the loss of relevant morphological information as root stemming affected the performance. Also, in^42^, different settings of LSTM hyper-parameters as batch size and output length, was tested using a large dataset of book reviews.

Combinations of CNN and LSTM were implemented to predict the sentiment of Arabic text in^43–46^. In a CNN–LSTM model, the CNN feature detector find local patterns and discriminating features and the LSTM processes the generated elements considering word order and context^46,47^. Most CNN-LSTM networks applied for Arabic SA employed one convolutional layer and one LSTM layer and used either word embedding^43,45,46^ or character representation^44^. Temporal representation was learnt for Arabic text by applying three stacked LSTM layers in^43^. The model performance was compared with CNN, one layer LSTM, CNN-LSTM and combined LSTM. Also, different optimizers were tested as Adam, Rmsprop, Adagrad and SGD. A worthy notice is that combining two LSTMs outperformed stacking three LSTMs due to the dataset size, as deep architectures require extensive data for feature detection.

Morphological diversity of the same Arabic word within different contexts was considered in a SA task by utilizing three types of feature representation^44^. Character, Character N-Gram, and word features were employed for an integrated CNN-LSTM model. The fine-grained character features enabled the model to capture more attributes from short text as tweets. The integrated model achieved an enhanced accuracy on the three datasets used for performance evaluation. Moreover, a hybrid dataset corpus was used to study Arabic SA using a hybrid architecture of one CNN layer, two LSTM layers and an SVM classifier^45^. The CNN-LSTM model was tested using one and two LSTM Layers. Stacked LSTM layers produced feature representations more appropriate for class discrimination. Various word embedding approaches were assessed. The results highlighted that the model realized the highest performance on the largest considered dataset. The online Arabic SA system Mazajak was developed based on a hybrid architecture of CNN and LSTM^46^. The model was evaluated on three benchmarking datasets. The applied word2vec word embedding was trained on a large and diverse dataset to cover several dialectal Arabic styles.

Bi-LSTM, the bi-directional version of LSTM, was applied to detect sentiment polarity in^47–49^. A bi-directional LSTM is constructed of a forward LSTM layer and a backward LSTM layer. The fore cells handle the input from start to end, and the back cells process the input from end to start. The two layers work in reverse directions, enabling to keep the context of both the previous and the following words^47,48^.

LSTM, Bi-LSTM and deep LSTM and Bi-LSTM with two layers were evaluated and compared for comments SA^47^. It was reported that Bi-LSTM showed more enhanced performance compared to LSTM. The deep LSTM further enhanced the performance over LSTM, Bi-LSTM, and deep Bi-LSTM. The authors indicated that the Bi-LSTM could not benefit from the two way exploration of previous and next contexts due to the unique characteristics of the processed data and the limited corpus size. Also, CNN and Bi-LSTM models were trained and assessed for Arabic tweets SA and achieved a comparable performance^48^. The separately trained models were combined in an ensemble of deep architectures that could realize a higher accuracy. In addition, The ability of Bi-LSTM to encapsulate bi-directional context was investigated in Arabic SA in^49^. CNN and LSTM were compared with the Bi-LSTM using six datasets with light stemming and without stemming. Results emphasized the significant effect of the size and nature of the handled data. The highest performance on large datasets was reached by CNN, whereas the Bi-LSTM achieved the highest performance on small datasets.

GRUs were studied in^14,50^ for Arabic sentiment identification. LSTM, Bi-LSTM, GRU, and Bi-GRU were used to predict the sentiment category of Arabic microblogs depending on Emojis features^14^. Results reported that Bi-GRU outperformed Bi-LSTM with slightly different performance on a small dataset of short dialectical Arabic tweets. Experiments evaluated diverse methods of combining the bi-directional features and stated that concatenation led to the best performance for LSTM and GRU. Besides, the detection of religious hate speech was analyzed as a classification task applying a GRU model and pre-trained word embedding^50^. The embedding was pre-trained on a Twitter corpus that contained different Arabic dialects. GRU outperformed other machine learning and lexicon-based classifiers. Supporting the GRU model with handcrafted features about time, content, and user boosted the recall measure.

A hybrid parallel model that utlized three seprate channels was proposed in^51^. The channels outputs were concatenated and fed to the final dense layer. Each channel is an independant model with a distinct input. Character CNN, word CNN, and sentence Bi-LSTM-CNN channels were trained parallel. A positioning binary embedding scheme (PBES) was proposed to formulate contextualized embeddings that efficiently represent character, word, and sentence features. The model was validated on 34 Arabic sentiment analysis datasets. Binary and tertiary hybrid datasets were also used for the model assessment. The model performance was more evaluated using the IMDB movie review dataset. Experimental results showed that the model outperformed the baselines for all datasets.

Another hybridization paradigm is combining word embedding and weighting techniques. Combinations of word embedding and weighting approaches were investigated for sentiment analysis of product reviews^52^. The embedding schemes Word2vec, GloVe, FastText, DOC2vec, and LDA2vec were combined with the TF-IDF, inverse document frequency, and smoothed inverse document frequency weighting approaches. To account for word relevancy, weighting approaches were used to weigh the word embedding vectors to account for word relevancy. Weighted sum, centre-based, and Delta rule aggregation techniques were utilized to combine embedding vectors and the computed weights. RNN, LSTM, GRU, CNN, and CNN-LSTM deep networks were assessed and compared using two Twitter corpora. The experimental results showed that the CNN-LSTM structure reached the highest performance. The LSTM network achieved the second-best performance.

Word embedding models such as FastText, word2vec, and GloVe were integrated with several weighting functions for sarcasm recognition^53^. Weighting mechanisms include TF-IDF, term-frequency, odds ratio, balanced distributional concentration, inverse gravity moment, short text weighting, regularized entropy, inverse false negative—true positive—inverse category frequency, relevance frequency, and inverse question frequency—question frequency-inverse category frequency were employed. The deep learning structures RNN, GRU, LSTM, Bi-LSTM, and CNN were used to classify text as sarcastic or not. Three sarcasm identification corpora containing tweets, quote responses, news headlines were used for evaluation. The proposed representation integrated word embedding, weighting functions, and N-gram techniques. The weighted representation of a document was computed as the concatenation of the weighted unigram, bigram and trigram representations. The three layers Bi-LSTM model trained with the trigrams of inverse gravity moment weighted embedding realized the best performance.

Combinations of word embedding and handcrafted features were investigated for sarcastic text categorization^54^. Sarcasm was identified using topic supported word embedding (LDA2Vec) and evaluated against multiple word embedding such as GloVe, Word2vec, and FastText. The CNN trained with the LDA2Vec embedding registered the highest performance, followed by the network that was trained with the GloVe embedding. Handcrafted features namely pragmatic, lexical, explicit incongruity, and implicit incongruity were combined with the word embedding. Diverse combinations of handcrafted features and word embedding were tested by the CNN network. The best performance was achieved by merging LDA2Vec embedding and explicit incongruity features. The second-best performance was obtained by combining LDA2Vec embedding and implicit incongruity features.

## Applied models

The hybrid notion was considered in SA by combining different features (word and character^22^; word and weighting techniques^52^; character, word, and sentence^51^), deep architectures (CNN and LSTM)^43–46^, approaches (lexicon-based and deep learning)^38,39^, and domains (video games reviews, cell phones reviews, and food reviews)^37^. Furthermore, different dialects were merged in the training corpus^22^. The proposed work applies multiple ways of hybridization namely hybrid deep architectures (CNN-LSTM, CNN-GRU, LSTM-CNN, GRU-CNN, CNN-Bi-LSTM, CNN-Bi-GRU, Bi-LSTM-CNN, and Bi-GRU-CNN), hybrid language styles (MSA and dialectical), and hybrid data sources (tweets, reviews) which proposes more challenges for Arabic SA. In addition, deep models based on a single architecture (LSTM, GRU, Bi-LSTM, and Bi-GRU) are also investigated. The datasets utilized to validate the applied architectures are a combined hybrid dataset and the Arabic book review corpus (BRAD).

### Network design

**Figure 1:**
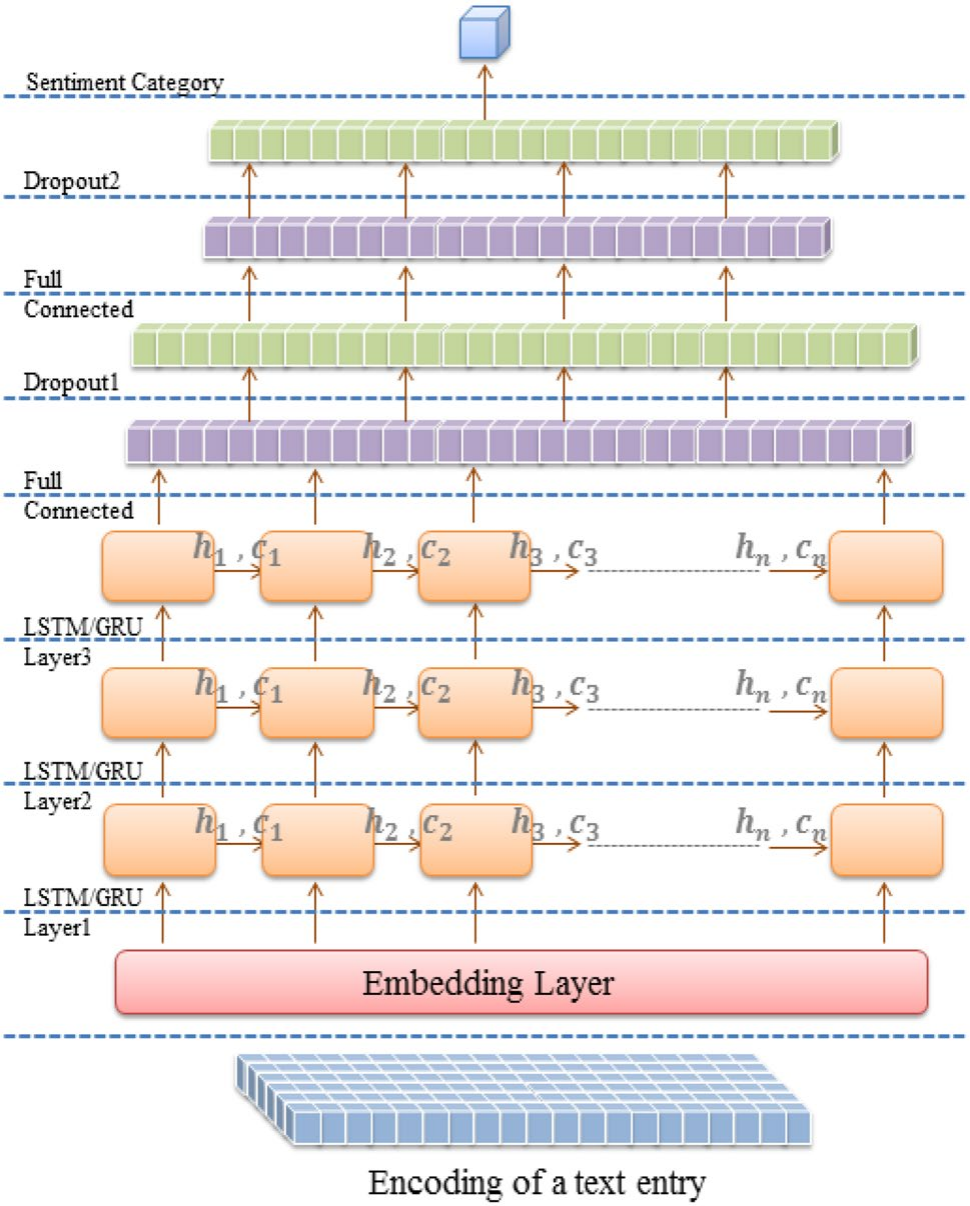
LSTM/GRU architecture (created by Microsoft PowerPoint 2010).

**Figure 2:**
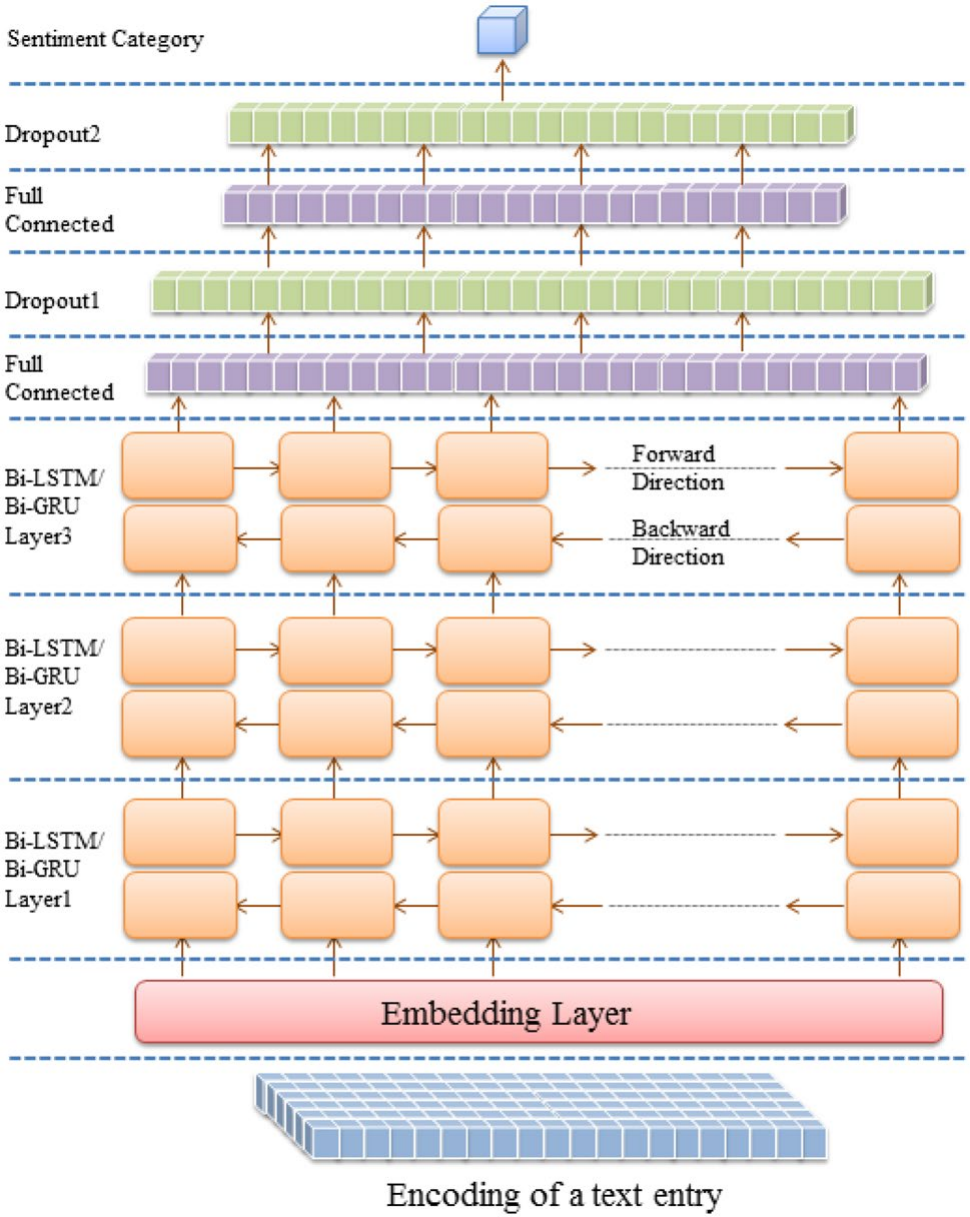
Bi-LSTM/Bi-GRU architecture (created by Microsoft PowerPoint 2010).

**Figure 3:**
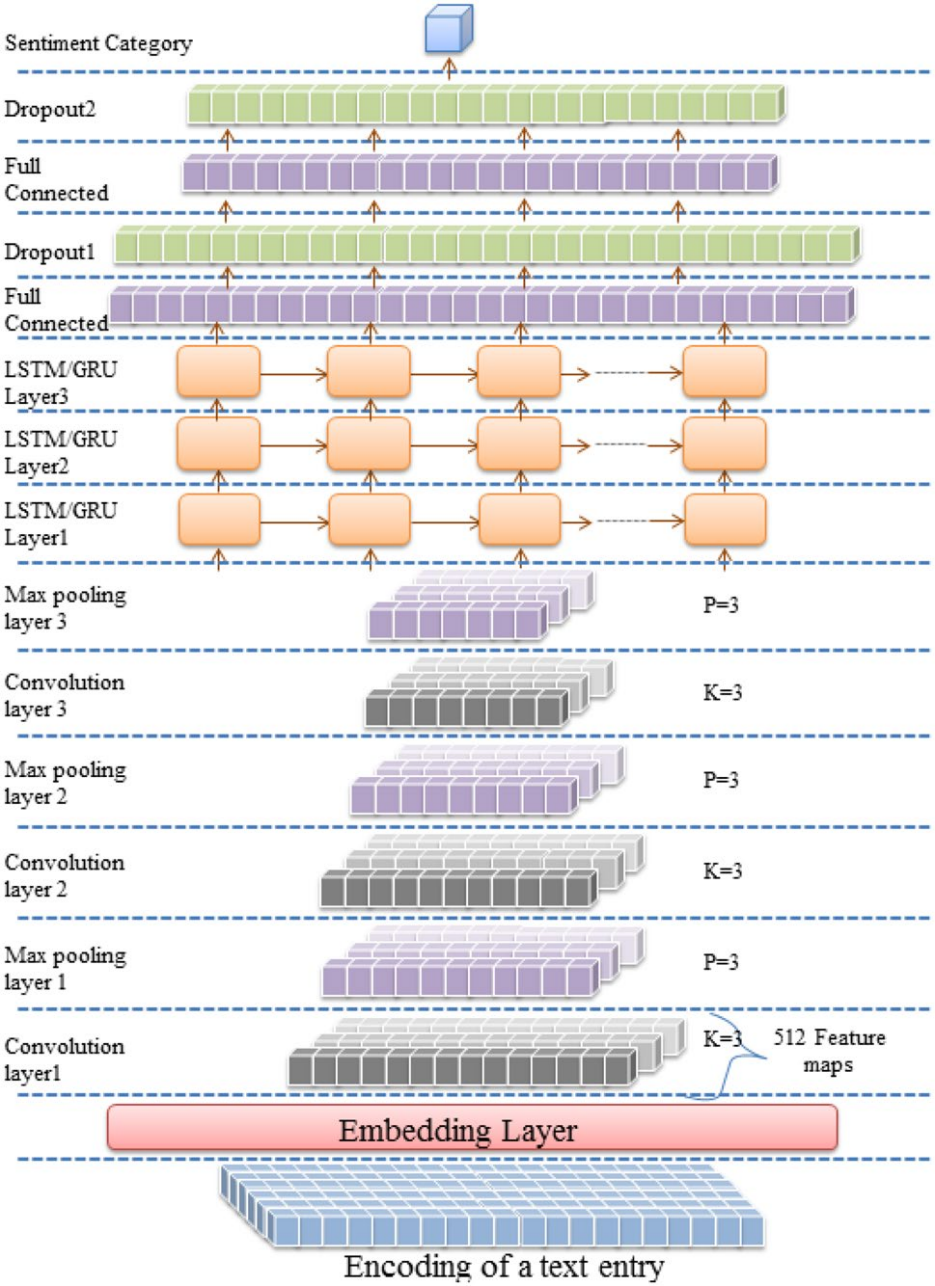
CNN-LSTM/CNN-GRU architecture (created by Microsoft PowerPoint 2010).

**Figure 4:**
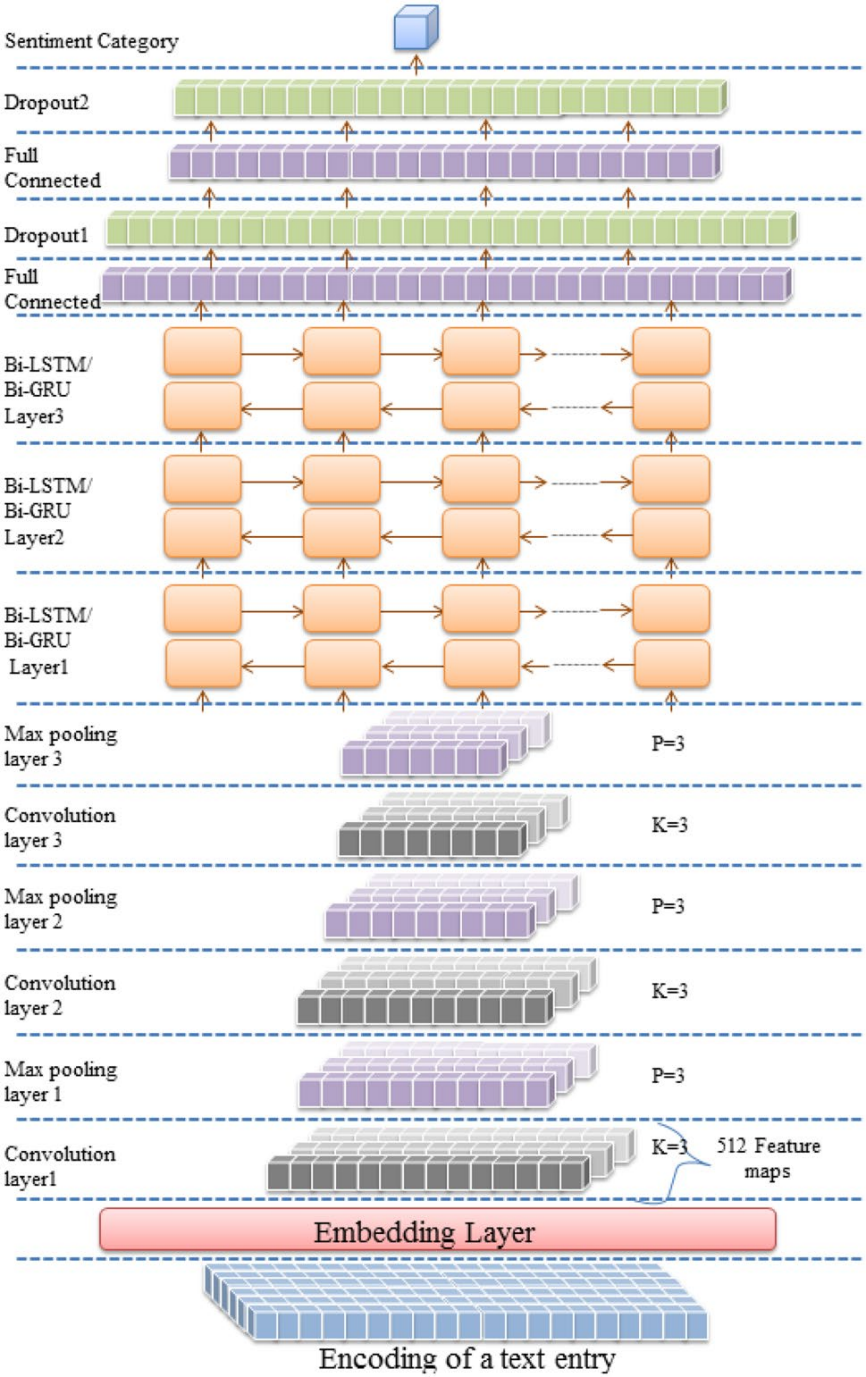
CNN-Bi-LSTM/CNN-Bi-GRU architecture (created by Microsoft PowerPoint 2010).

Multiple deep architectures are implemented for Arabic SA. All architectures employ a character embedding layer to convert encoded text entries to a vector representation. Feature detection is conducted in the first architecture by three LSTM, GRU, Bi-LSTM, or Bi-GRU layers, as shown in Figs. 1 and 2. The discrimination layers are three fully connected layers with two dropout layers following the first and the second dense layers. In the dual architecture, feature detection layers are composed of three convolutional layers and three max-pooling layers arranged alternately, followed by three LSTM, GRU, Bi-LSTM, or Bi-GRU layers. Finally, the hybrid layers are mounted between the embedding and the discrimination layers, as described in Figs. 3 and 4.

### Network settings

Each opinion entry is represented as a sequence of characters. The character vocabulary includes all characters found in the dataset (Arabic characters, , Arabic numbers, English characters, English numbers, emoji, emoticons, and special symbols). A vocabulary set of (746) characters is used for encoding the text corpus. Opinion entries are quantized as sequences of length 1014 characters. The training methodology and settings are conducted following^27,55^. Python, Keras, and Tensorflow are used for the models application. CNN, LSTM, GRU, Bi-LSTM, and Bi-GRU layers are trained on CUDA11 and CUDNN10 for acceleration. The implementation is conducted on NVIDIA GEFORCE GTX 1070 GPU. The settings of the applied architectures are stated in Tables 2 and 3.

**Table 2:** Network settings for feature detection layers.

Architecture	Parameter	Settings
Gated RNNs	Embedding size	16
	LSTM, GRU, Bi-LSTM, Bi-GRU layers	3
	LSTM, GRU cells	100 in each layer
	Bi-LSTM, Bi-GRU cells	100 in each direction
Gated RNNs—CNN	Embedding size	16
	LSTM, GRU, Bi-LSTM, Bi-GRU layers	3
	LSTM, GRU cells	100 in each layer
	Bi-LSTM, Bi-GRU cells	100 in each direction
	CNN layers	3
	CNN local receptive field (kernel)	3
	CNN feature maps	512
	Pooling layers	3
	Pooling size	3
CNN—gated RNNs	Embedding size	16
	CNN layers	3
	CNN local receptive field (kernel)	3
	CNN feature maps	512
	Pooling layers	3
	Pooling size	3
	LSTM, GRU, Bi-LSTM, Bi-GRU layers	3
	LSTM, GRU cells	100 in each layer
	Bi-LSTM, Bi-GRU cells	100 in each direction

**Table 3:** Network settings for discrimination layers.

Parameter	Settings
Dense layer one cells	2048
Dropout 1	0.5
Dense layer two cells	2048
Dropout 2	0.5
Dense layer three cells	1

## Experiments and results

### Data preparation and preprocessing

Two datasets are used for training and testing the described architectures. The first dataset is a hybrid dataset built from ten free accessible Arabic sentiment analysis corpora. Opinion entries are composed in colloquial and modern standard Arabic and belong to various domains: tweets, product reviews, restaurant reviews, hotel reviews, book reviews, and movie reviews. Only positive and negative categories are used to build the training set. The combined, balanced and hybrid dataset contains (146,388) samples. Table 4 describes the corpora used to construct the mixed dataset.

**Table 4:** The Arabic sentiment analysis corpora.

Name	Total entries	Domain	Positive entries	Negative entries
^59^ TDS	2000	Tweeter	1000	1000
^60^ ASTD	10,006	Tweeter	799	1684
^61^ SemEval	671	Tweeter	222	128
^62^ Social media posts	3200	Tweeter/blogs	719	1760
^63^ OCA	500	Movie reviews	250	250
^64^ LABR	63,257	Book reviews	42,832	8224
^65^ LARGE	34,492	Reviews	24,948	6650
^66^ Health services	2026	Tweeter	628	1398
^67^ HARD	409,562	Hotel reviews	52,849	52,849
^68^ ArSAS	21,064	Tweeter	4643	7840

The second dataset is BRAD, a publicly available corpus for Arabic sentiment analysis^56^. BRAD was collected from “http://www.goodreads.com” and includes (510,598) book reviews. The balanced dataset contains (156,506) samples. Reviews are composed in modern standard and colloquial Arabic. Books were rated on a scale from 1 to 5 where ratings 4 and 5 belong to the positive category and ratings 1 and 2 belong to the negative category. For both sets, 70% of the samples are reserved for training, 20% are used for development, and 10% are employed for testing.

### Results analysis

The measures used to evaluate the efficiency of the applied models are accuracy and F-score. Accuracy is the percentage of correctly predicted samples. F-score is the harmonic mean of precision and recall^34^. Precision is the ratio between the correctly predicted positive entries to all the entries that are predicted as positive. The recall is the ratio between the correctly predicted positive entries to all the entries that are real positive. The performance measures indicate the ability of the deep models to discriminate both polarity categories. Equations (1), (2), (3) and (4) identify how the estimates are calculated^69^:

1 $${\text{Precision }} = \, \left( {{\text{TP}}/ \, \left( {{\text{TP}} + {\text{ FP}}} \right)} \right)$$

2 $${\text{Recall }} = \, \left( {{\text{TP}}/ \, \left( {{\text{TP}} + {\text{ FN}}} \right)} \right)$$

3 $${\text{Accuracy }} = \, \left( {\left( {{\text{TP}} + {\text{TN}}} \right) \, / \, \left( {{\text{TP}} + {\text{TN}} + {\text{ FP}} + {\text{FN}}} \right)} \right)$$

4 $${\text{F-score }} = \left( {\left( {{\text{Precision }} \times {\text{ Recall}}} \right) \, / \, \left( {{\text{Precision }} + {\text{ Recall}}} \right)} \right) \, \times {2}$$

where: TP is the number of true-positive instances, TN is the number of true-negative instances, FP is the number of false-positive instances, FN is the number of false-negative instances.

To mitigate bias and preserve the text semantics no extensive preprocessing as stemming, normalization, and lemmatization is applied to the datasets, and the considered vocabulary includes all the characters that appeare in the dataset^57,58^. Also, all terms in the corpus are encoded, including stop words and Arabic words composed in English characters that are commonly removed in the preprocessing stage. The elimination of such observations may influence the understanding of the context.

GRU models showed higher performance based on character representation than LSTM models. Although the models share the same structure and depth, GRUs learned and disclosed more discriminating features. On the other hand, the hybrid models reported higher performance than the one architecture model. Employing LSTM, GRU, Bi-LSTM, and Bi-GRU in the initial layers showed more boosted performance than using CNN in the initial layers. In addition, bi-directional LSTM and GRU registered slightly more enhanced performance than the one-directional LSTM and GRU.

Comparing the performance of the trained models using the hybrid dataset indicates that the Bi-GRU-CNN model achieved the highest accuracy, 89.67, followed by the GRU-CNN model with 89.65% as stated in Table 5. The Bi-LSTM model registered the least accuracy with 87.85. The highest LSTM accuracy is 89.30% achieved by the Bi-LSTM-CNN model, and the lowest accuracy is 88.12 reported by the CNN-LSTM model. Results show that starting the models with CNN layers is not beneficial for detecting efficient features.

**Table 5:** Comparison of applied models’ performance on the hybrid dataset.

Architecture	Precision	Recall	F-score	Accuracy
LSTM	88.89	88.85	88.84	88.85
GRU	88.98	88.92	88.92	88.92
LSTM-CNN	88.82	88.79	88.79	88.79
GRU-CNN	**89.66**	**89.65**	**89.65**	**89.65**
CNN-LSTM	88.13	88.12	88.12	88.12
CNN-GRU	88.11	88.10	88.10	88.10
Bi-LSTM	88.03	87.85	87.83	87.85
Bi-GRU	88.96	88.85	88.84	88.85
Bi-LSTM-CNN	89.31	89.30	89.30	89.30
Bi-GRU-CNN	**89.69**	**89.67**	**89.66**	**89.67**
CNN-Bi-LSTM	88.52	88.51	88.51	88.51
CNN-Bi-GRU	88.20	88.17	88.17	88.17

Significant values are in given in bold.

The Bi-GRU-CNN model showed the highest performance with 83.20 accuracy for the BRAD dataset, as reported in Table 6. In addition, the model achived nearly 2% improved accuracy compared to the Deep CNN ArCAR System^21^ and almost 2% enhanced F-score, as clarified in Table 7. The GRU-CNN model registered the second-highest accuracy value, 82.74, with nearly 1.2% boosted accuracy. Also, the LSTM model with 82.14 increased the accuracy by almost 0.7%.

**Table 6:** Comparison of applied models’ performance on the BRAD data set.

Architecture	Precision	Recall	F-score	Accuracy
LSTM	82.16	82.14	82.14	82.14
GRU	80.62	80.62	80.61	80.61
LSTM-CNN	80.24	79.90	79.85	79.91
GRU-CNN	**82.74**	**82.74**	**82.74**	**82.74**
CNN-LSTM	80.69	80.69	80.68	80.68
CNN-GRU	80.81	80.81	80.81	80.81
Bi-LSTM	81.14	81.13	81.13	81.13
Bi-GRU	81.18	81.18	81.18	81.18
Bi-LSTM-CNN	80.09	80.07	80.07	80.07
Bi-GRU-CNN	**83.22**	**83.20**	**83.20**	**83.20**
CNN-Bi-LSTM	80.89	80.89	80.89	80.89
CNN-Bi-GRU	80.44	80.44	80.44	80.44

Significant values are in given in bold.

**Table 7:** Comparison of Bi-GRU-CNN performance and the related literature on the BRAD dataset.

Architecture	Precision	Recall	F-score	Accuracy
^21^ Deep CNN ArCAR system	81.48	81.44	81.45	81.46
Bi-GRU-CNN	83.22	83.20	83.20	83.20
GRU-CNN	82.74	82.74	82.74	82.74
LSTM	82.16	82.14	82.14	82.14

Another experiment was conducted to evaluate the ability of the applied models to capture language features from hybrid sources, domains, and dialects. The models trained on the mixed dataset are tested using the BRAD test set. The Bi-GRU-CNN model reported the highest performance on the BRAD test set, as shown in Table 8. The hybrid model can correctly classify nearly 76% of the test set. Results prove that the knowledge learned from the hybrid dataset can be exploited to classify samples from unseen datasets. The exhibited performace is a consequent on the fact that the unseen dataset belongs to a domain already included in the mixed dataset. Using a giant hybrid dataset can increase the model capability.

**Table 8:** Performance of models trained on the Hybrid dataset and tested using the BRAD test set.

Architecture	Precision	Recall	F-score	Accuracy
LSTM	75.42	71.35	70.13	71.32
GRU	75.02	70.94	69.67	70.90
LSTM-CNN	77.03	74.03	73.28	74.01
GRU-CNN	77.29	74.68	74.05	74.66
CNN-LSTM	74.88	71.49	70.46	71.46
CNN-GRU	75.08	71.76	70.77	71.73
Bi-LSTM	72.41	65.97	63.30	65.93
Bi-GRU	74.14	68.97	67.18	68.93
Bi-LSTM-CNN	77.43	74.19	73.38	74.16
Bi-GRU-CNN	**78.67**	**75.99**	**75.39**	**75.96**
CNN-Bi-LSTM	75.42	72.16	71.22	72.13
CNN-Bi-GRU	74.96	71.70	70.72	71.67

Significant values are in given in bold.

**Figure 5:**
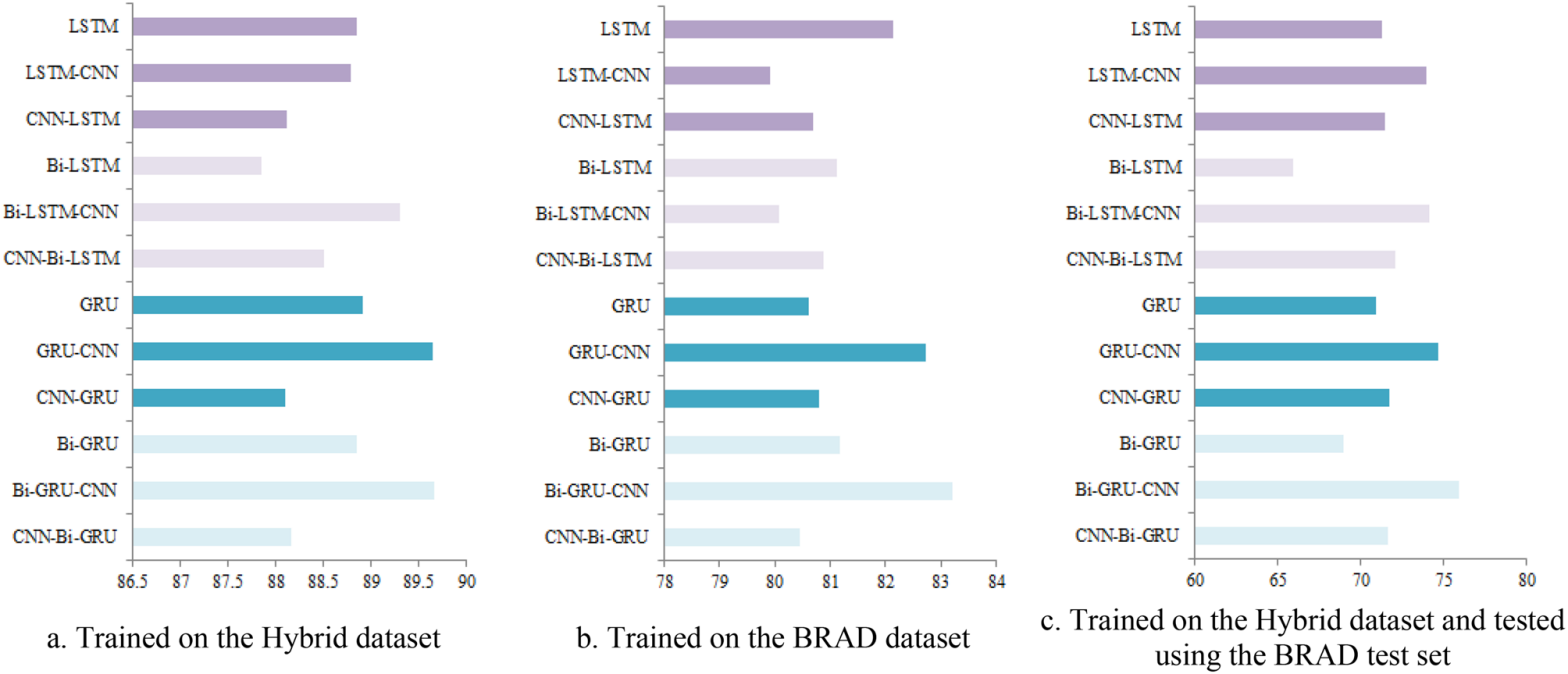
Accuracy of LSTM/GRU based architectures (created by Microsoft PowerPoint 2010).

The accuracy of the LSTM based architectures versus the GRU based architectures is illastrated in Fig. 5. Results show that GRUs are more powerful to disclose features from the rich hybrid dataset. On the other hand, LSTMs are more sensitive to the nature and size of the manipulated data. Stacking multiple layers of CNN after the LSTM, GRU, Bi-GRU, and Bi-LSTM reduced the number of parameters and boosted the performance. The networks parameters are listed in Table 9.

**Table 9:** The networks parameers.

Architecture	Total params (M)
LSTM	212.088
GRU	212.036
LSTM-CNN	43.905
GRU-CNN	43.853
CNN-LSTM	13.599
CNN-GRU	13.497
Bi-LSTM	420.124
Bi-GRU	419.980
Bi-LSTM-CNN	44.429
Bi-GRU-CNN	44.284
CNN-Bi-LSTM	21.540
CNN-Bi-GRU	21.296

Precision, Recall, and F-score of the trained networks for the positive and negative categories are reported in Tables 10 and 11. The inspection of the networks performance using the hybrid dataset indicates that the positive recall reached 0.91 with the Bi-GRU and Bi-LSTM architectures. Considering the positive category the recall or sensitivity measures the network ability to discriminate the actual positive entries^69^. The precision or confidence which measures the true positive accuracy registered 0.89 with the GRU-CNN architecture. Similar statistics for the negative category are calculated by predicting the opposite case^70^. The negative recall or specificity evaluates the network identification of the actual negative entries registered 0.89 with the GRU-CNN architecture. The negative precision or the true negative accuracy, which estimates the ratio of the predicted negative samples that are really negative, reported 0.91 with the Bi-GRU architecture.

**Table 10:** Performance measures of the hybrid dataset.

	Precision	Recall	F-score		Precision	Recall	F-score
**a. LSTM**				**b. GRU**			
Negative	0.9024	0.8711	0.8865	Negative	0.9050	0.8698	0.8870
Positive	0.8755	0.9058	0.8904	Positive	0.8747	0.9086	0.8913
Average	0.8889	0.8885	0.8884	Average	0.8898	0.8892	0.8892
**c. LSTM-CNN**				**d. GRU-CNN**			
Negative	0.8986	0.8746	0.8864	Negative	0.9014	0.8904	0.8959
Positive	0.8778	0.9013	0.8894	Positive	0.8918	0.9026	0.8972
Average	0.8882	0.8879	0.8879	Average	0.8966	0.8965	0.8965
**e. CNN-LSTM**				**f. CNN-GRU**			
Negative	0.8891	0.8710	0.8799	Negative	0.8863	0.8742	0.8802
Positive	0.8736	0.8913	0.8824	Positive	0.8759	0.8879	0.8818
Average	0.8813	0.8812	0.8812	Average	0.8811	0.8810	0.8810
**g. Bi-LSTM**				**h. Bi-GRU**			
Negative	0.9070	0.8434	0.8740	Negative	0.9108	0.8613	0.8854
Positive	0.8536	0.9136	0.8826	Positive	0.8685	0.9157	0.8914
Average	0.8803	0.8785	0.8783	Average	0.8896	0.8885	0.8884
**i. Bi-LSTM-CNN**				**j. Bi-GRU-CNN**			
Negative	0.8999	0.8844	0.8921	Negative	0.9061	0.8850	0.8954
Positive	0.8864	0.9016	0.8939	Positive	0.8876	0.9083	0.8978
Average	0.8931	0.8930	0.8930	Average	0.8969	0.8967	0.8966
**k. CNN-Bi-LSTM**				**l. CNN-Bi-GRU**			
Negative	0.8929	0.8752	0.8839	Negative	0.8914	0.8693	0.8802
Positive	0.8776	0.8950	0.8862	Positive	0.8725	0.8941	0.8832
Average	0.8852	0.8851	0.8851	Average	0.8820	0.8817	0.8817

**Table 11:** Performance measures of the BRAD dataset.

	Precision	Recall	F-score		Precision	Recall	F-score
**a. LSTM**				**b. GRU**			
Negative	0.8137	0.8345	0.8240	Negative	0.8084	0.8033	0.8058
Positive	0.8296	0.8083	0.8188	Positive	0.8039	0.8090	0.8065
Average	0.8216	0.8214	0.8214	Average	0.8062	0.8062	0.8061
**c. LSTM-CNN**				**d. GRU-CNN**			
Negative	0.7709	0.8519	0.8094	Negative	0.8305	0.8234	0.8270
Positive	0.8339	0.7461	0.7875	Positive	0.8244	0.8314	0.8279
Average	0.8024	0.7990	0.7985	Average	0.8274	0.8274	0.8274
**e. CNN-LSTM**				**f. CNN-GRU**			
Negative	0.8097	0.8030	0.8064	Negative	0.8129	0.8011	0.8070
Positive	0.8040	0.8107	0.8073	Positive	0.8033	0.8151	0.8091
Average	0.8069	0.8069	0.8068	Average	0.8081	0.8081	0.8081
**g. Bi-LSTM**				**h. Bi-GRU**			
Negative	0.8086	0.8165	0.8125	Negative	0.8116	0.8128	0.8122
Positive	0.8141	0.8061	0.8101	Positive	0.8119	0.8107	0.8113
Average	0.8114	0.8113	0.8113	Average	0.8118	0.8118	0.8118
**i. Bi-LSTM-CNN**				**j. Bi-GRU-CNN**			
Negative	0.8095	0.7873	0.7983	Negative	0.8401	0.8207	0.8303
Positive	0.7924	0.8142	0.8031	Positive	0.8242	0.8433	0.8337
Average	0.8009	0.8007	0.8007	Average	0.8322	0.8320	0.8320
**k. CNN-Bi-LSTM**				**l. CNN-Bi-GRU**			
Negative	0.8104	0.8072	0.8088	Negative	0.8066	0.8017	0.8041
Positive	0.8074	0.8106	0.8090	Positive	0.8023	0.8071	0.8047
Average	0.8089	0.8089	0.8089	Average	0.8044	0.8044	0.8044

On the other side, for the BRAD dataset the positive recall reached 0.84 with the Bi-GRU-CNN architecture. The precision or confidence registered 0.83 with the LSTM-CNN architecture. The negative recall or Specificity acheived 0.85 with the LSTM-CNN architecture. The negative precision or the true negative accuracy reported 0.84 with the Bi-GRU-CNN architecture. The confusion matrices of the networks are stated in Tables 12 and 13. In some cases identifying the negative category is more significant than the postrive category, especially when there is a need to tackle the issues that negatively affected the opinion writer. In such cases the candidate model is the model that efficiently discriminate negative entries.

**Table 12:** Confusion matrices of the hybrid dataset.

	Predicted label			Predicted label	
True label	Negative	Positive	True label	Negative	Positive
**a. LSTM**			**b. GRU**		
Negative	7085	1048	Negative	7074	1059
Positive	766	7367	Positive	743	7390
**c. LSTM-CNN**			**d. GRU-CNN**		
Negative	7113	1020	Negative	7242	891
Positive	803	7330	Positive	792	7341
**e. CNN-LSTM**			**f. CNN-GRU**		
Negative	7084	1049	Negative	7110	1023
Positive	884	7249	Positive	912	7221
**g. Bi-LSTM**			**h. Bi-GRU**		
Negative	6859	6859	Negative	7005	1128
Positive	703	7430	Positive	686	7447
**i. Bi-LSTM-CNN**			**j. Bi-GRU-CNN**		
Negative	7193	940	Negative	7198	935
Positive	800	7333	Positive	746	7387
**k. CNN-Bi-LSTM**			**l. CNN-Bi-GRU**		
Negative	7118	1015	Negative	7070	1063
Positive	854	7279	Positive	861	7272

**Table 13:** Confusion matrices of the BRAD dataset.

	Predicted label			Predicted label	
True label	Negative	Positive	True label	Negative	Positive
**a. LSTM**			**b. GRU**		
Negative	6541	1297	Negative	6296	1542
Positive	1498	6315	Positive	1492	6321
**c. LSTM-CNN**			**d. GRU-CNN**		
Negative	6677	1161	Negative	6454	1384
Positive	1984	5829	Positive	1317	6496
**e. CNN-LSTM**			**f. CNN-GRU**		
Negative	6294	1544	Negative	6279	1559
Positive	1479	6334	Positive	1445	6368
**g. Bi-LSTM**			**h. Bi-GRU**		
Negative	6400	1438	Negative	6371	1467
Positive	1515	6298	Positive	1479	6334
**i. Bi-LSTM-CNN**			**j. Bi-GRU-CNN**		
Negative	6171	1667	Negative	6433	1405
Positive	1452	6361	Positive	1224	6589
**k. CNN-Bi-LSTM**			**l. CNN-Bi-GRU**		
Negative	6327	1511	Negative	6284	1554
Positive	1480	6333	Positive	1507	6306

## Conclusion

Deep neural architectures have proved to be efficient feature learners, but they rely on intensive computations and large datasets. In the proposed work, LSTM, GRU, Bi-LSTM, Bi-GRU, and CNN were investigated in Arabic sentiment polarity detection. Character features are used to encode the morphology and semantics of text. The applied models showed a high ability to detect features from the user-generated text. The model layers detected discriminating features from the character representation. GRU models reported more promoted performance than LSTM models with the same structure.

Moreover, deep hybrid networks realized the highest performance measures. Combining LSTM, GRU, Bi-LSTM, and Bi-GRU with CNN boosted the performance. Bi-GRU-CNN hybrid models registered the highest accuracy for the hybrid and BRAD datasets. On the other hand, the Bi-LSTM and LSTM-CNN models wrote the lowest performance for the hybrid and BRAD datasets. The proposed Bi-GRU-CNN model reported 89.67% accuracy for the mixed dataset and nearly 2% enhanced accuracy for the BRAD corpus.

In addition, the Bi-GRU-CNN trained on the hyprid dataset identified 76% of the BRAD test set. Therefore, hybrid models that combine different deep architectures can be implemented and assessed in different NLP tasks for future work. Also, the performance of hybrid models that use multiple feature representations (word and character) may be studied and evaluated.
